# Differential effects of inspiratory muscle training on diaphragm thickness and exercise tolerance in childhood cancer survivors and non-cancer controls

**DOI:** 10.3389/fped.2026.1810953

**Published:** 2026-04-10

**Authors:** Simon Ho, Victoria Marchese

**Affiliations:** Department of Physical Therapy and Rehabilitation Science, University of Maryland School of Medicine, Baltimore, MD, United States

**Keywords:** 6-minute walk test, diaphragm, exertional dyspnea, pediatric oncology, ultrasonography

## Abstract

Despite major advances in cancer treatment, the majority of childhood cancer survivors (CCS) will continue to experience cancer treatment-related side effects. Pulmonary dysfunction is a major late effect that can be worsened by respiratory muscle dysfunction. Inspiratory muscle training (IMT) is a possible solution, but CCS may be less responsive to training compared to healthy, typically developing, non-cancer controls (HTD). In this exploratory study, we investigated the effects of 6-weeks of IMT [5 sets of 6 breaths, 5 days per week, at 75% of maximal inspiratory pressure (MIP)] in a home-based setting with weekly monitoring in 7 CCS and 10 HTD, aged between 6 and 12 years. Diaphragm structure and function as well as exercise tolerance were measured before (T1) and after training (T2). Diaphragm thickness at total lung capacity, measured via ultrasonography, improved in CCS [T1 vs. T2: +0.05, 95% confidence interval: (0.01, 0.1) cm, *P* = 0.02], but not in HTD. Whereas chest wall excursion (CWE) and performance on the 6-minute walk test (*z*-score) improved in HTD [T1 vs. T2 CWE: +0.81 (0.23, 1.39) cm, *P* < 0.001; T1 vs. T2 6MWT *z*-score: +0.49 (0.23, 0.75), *P* < 0.001], but not in CCS. Respiratory muscle performance, measured by MIP (% predicted) and muscle quality, defined as the ratio of MIP (cmH_2_O) to diaphragm thickness, improved in both groups (*P* < 0.05 for all). Thus, we provide preliminary findings suggesting that there might have been differences in the response to training due to cancer treatment-related late effects in CCS. However, these findings should be interpreted with caution given the exploratory nature of our study. Nonetheless, the overall positive benefits of IMT on the respiratory muscles support its potential clinical utility in CCS.

**Clinical Trial Registration:**

http://ClinicalTrials.gov NCT06205251 and NCT06230692.

## 1 Introduction

In recent decades, major strides in the treatment of childhood cancers have improved the 5-year survival rate to over 85% (1). However, the majority of childhood cancer survivors (CCS) will continue to experience cancer treatment-related side effects, affecting virtually all organ systems (2). Pulmonary dysfunction is a common late effect that often goes undetected (3). In particular, CCS who received allogenic hematopoietic stem cell transplant, thoracic radiation, or thoracic surgery are at the highest risk (4). Exposure to specific chemotherapeutic agents such as bleomycin, busulfan, nitrosoureas, cyclophosphamide, and methotrexate also elevate risk (4, 5). Furthermore, muscle dysfunction is another common late effect in CCS (6), especially for those who were exposed to anthracyclines (7, 8). Specifically for breathing, impaired muscle function is of great concern because of the risk to the respiratory muscles (9), which can worsen pulmonary dysfunction. As such, together or on their own, these late effects carry major implications for the development of exertional dyspnea and exercise intolerance (10, 11).

To date, little is known about effective treatments for pulmonary or respiratory muscle-specific late effects in CCS. Whole-body, exercise training shows promise in improving overall exercise tolerance and physical function (12, 13). Yet, the impact of exercise training on pulmonary function or the respiratory muscles has not been directly studied. This is an important gap in knowledge because these specific late effects, if not addressed, will likely persist and increase the risk of morbidity and mortality later in life (4). Inspiratory muscle training (IMT), a type of resistance exercise, is a possible solution with demonstrated clinical benefits in pulmonary function and respiratory muscle strength in children (14), but not much is known about this type of training in CCS. Whether pulmonary late effects can be addressed by IMT in CCS is not clear.

Because of the various late effects that might affect typical growth and development (15–17), CCS may not respond as expected to IMT. The risk of pulmonary dysfunction, chest wall restrictions, and stunted growth all could contribute to limited improvements in flow and volume. Furthermore, the risk of muscle dysfunction makes the diaphragm, the primary muscle of respiration, susceptible to injury, leading to adverse changes in structural and contractile properties (18). As such, the usual coupling of diaphragm and chest wall mechanics is likely disrupted (19), potentially affecting the responsiveness of IMT in CCS. Making matters worse, exposure to anthracyclines increases the risk of impairing muscle satellite cells, which are required for protein synthesis and muscle regeneration, thus making hypertrophy more difficult (20, 21). Nonetheless, in a study of adult survivors of lung and breast cancer, who are at risk for the same late effects, IMT improved overall respiratory muscle strength and exercise tolerance (22). Thus, IMT is promising for CCS in spite of disadvantages owing to various late effects. However, there might be differential responses to training when compared to a group of non-cancer controls.

Thus, the goal of this exploratory study was to better understand the specific effects of IMT on diaphragm structure and function as well as exercise tolerance in CCS. Because of the aforementioned risk factors, we hypothesized that CCS will be less responsive to IMT compared to a group of healthy, typically developing, non-cancer controls (HTD). As such, our hypothesis was threefold: 1) diaphragm thickness will improve after IMT in HTD, but not in CCS; 2) respiratory muscle performance will improve after IMT in both groups; and 3) exercise tolerance will improve in both groups.

## 2 Methods

### 2.1 Participants

Seven CCS (4 females, 3 males) and 10 HTD (5 females, 5 males) were included in this study. For both groups, the eligibility criteria were: 1) age between 6 and 12 years; 2) no history of neuromuscular, cardiac, or pulmonary disease; and 3) no recent injury or condition (less than 6 months prior) that would preclude strength testing or participation in exercise. Additional criteria for the CCS group were: 1) completion of medical treatment any type of cancer, except for cancer of the central nervous system and 2) at least 1 year has passed since completion of medical treatment before participating in the study.

### 2.2 Study overview

This study used a pre-test/post-test design to determine the effects of IMT in 2 cohorts, CCS and HTD. Participants presented to the laboratory for baseline testing (T1), followed by 6 weeks of IMT at home with once weekly virtual follow-up to support training, and finally post-intervention testing (T2). At T1 and T2 sessions, study participants underwent the same series of tests and measures. During each testing session, participants were accompanied by a parent/caregiver, who provided written, informed consent. First, height in cm and weight in kg were measured on a standard stadiometer and digital scale, respectively, with shoes off. This was followed by measurement of spirometry, respiratory muscle strength, chest wall excursion, diaphragm thickness and excursion via ultrasonography, and finally exercise testing. The study protocol was approved by the local Institutional Review Board and was registered on ClinicalTrials.gov (NCT06205251 and NCT06230692).

### 2.3 Spirometry

Spirometry was assessed using the MGC Diagnostics Ultima PFX System (St. Paul, MN, USA) following standard procedures per published guidelines from the European Respiratory Society (ERS)/American Thoracic Society (ATS) (23). Participants were instructed to wear a disposable nose clip and to wrap their mouths around a mouthpiece with a viral and bacterial filter. To assess forced vital capacity (FVC), participants were asked to take a deep breath to total lung capacity (TLC), or maximal inhalation, followed by a forced expiratory maneuver, continued expiration until no more air can be expelled, and finally inspiration back to TLC. Trials were repeated until 3 acceptable maneuvers had been performed according to recommendations from the ATS.

### 2.4 Respiratory muscle strength

Maximal inspiratory pressure (MIP) in cmH_2_O was assessed using the MGC Diagnostics Ultima PFX System (St. Paul, MN, USA) according to ERS/ATS standards (24). Participants were instructed to wear a disposable nose clip and to wrap their mouths around a flanged mouthpiece with a viral and bacterial filter while they performed an inspiratory maneuver for 1.5 to 3 s. Participants performed each maneuver in a seated position starting with the lungs at residual volume (RV) for MIP. One-minute rest breaks were given between trials. Trials were repeated until three acceptable trials of the inspiratory maneuver that varied by less than 10% had been performed.

### 2.5 Chest wall excursion

Chest wall excursion (CWE) was measured using a tape measure at the level of the xiphoid process. Participants were asked to exhale completely to RV followed by a deep breath to TLC. The change in circumference from RV to TLC in cm was recorded as the excursion. Each participant performed a total of three trials.

### 2.6 Diaphragm thickness

Ultrasound images of diaphragm thickness were obtained using the Philips Affiniti 70 Ultrasound System with the eL18-4 linear transducer (Philips Healthcare, Cambridge, MA) via two-dimensional B-mode as previously described (25). Briefly, participants were first placed in a supine position. The transducer was then placed on the right lateral chest wall at the zone of apposition of the diaphragm, which was defined by the anterior and mid-axillary lines between ribs 7 and 10. Images were obtained with the transducer on the 8th intercostal space using the liver as an acoustic window to visualize the right hemidiaphragm. Images for thickness at maximal inspiration were obtained from three deep breaths at TLC. Images for thickness at rest were obtained from three quiet breaths at functional residual capacity (FRC). To ensure that thickness could be measured at approximately the same location of the costal diaphragm throughout the respiratory cycle, images of deep breaths were always obtained before images of quiet breaths. This was done because costal fibers that are closer to the dome of the diaphragm are more likely to become obscured by the lungs with deep breaths. Thus, the costal fibers that are still visible at TLC were used as a reference to measure thickness at both TLC and FRC.

Images were processed offline on FIJI/ImageJ (National Institutes of Health, Bethesda, MD). Thickness was defined as the perpendicular distance from the middle of the pleural line, representing the diaphragmatic pleura, to the middle of the peritoneal line, representing the peritoneal membrane. More specifically, this distance was measured inferior to the costophrenic angle, that is, the point where the costal diaphragm “peels away” from the chest wall during inspiration. As such, measurements were obtained at a point where the pleural and peritoneal lines were roughly parallel and the diaphragm was still approximating the chest wall. Thickening fraction (TF) was used to represent the change in thickness during deep breaths: TF at TLC=(Thickness at TLC - Thickness at FRC)/Thickness at FRC. Reliability of measuring diaphragm thickness via ultrasonography [intra-class correlation coefficients (ICC): intra-rate*r* = 0.97; inter-rate*r* = 0.92] was demonstrated in a previously published study (25).

### 2.7 Diaphragm excursion

Ultrasound images of diaphragm excursion were obtained using the Philips Affiniti 70 Ultrasound System with the C5-1 curvilinear transducer (Philips Healthcare, Cambridge, MA) via two-dimensional M-mode as previously described (25). Briefly, participants were first placed in a supine position. The probe was then placed on the right subcostal area below the costal margin near the midclavicular line and was directed cranially and dorsally so that the ultrasound beam was perpendicular to the posterior third of the diaphragm. In M-mode, the diaphragm can be visualized as an echogenic tracing line that moves towards the probe with inspiration. The liver was used as an acoustic window to visualize the right hemidiaphragm. Excursion was measured by tracking the movement of the tracing line with inspiration. Measurements for excursion in cm, duration in s, and velocity in cm/s were obtained for three quiet breaths to tidal volume (Vt), three deep breaths to TLC, and three sniff maneuvers (SNF). The sniff maneuver, a strong and sharp inspiratory effort through the nose, was used to assess for overall voluntary respiratory muscle activation, which required coordination of the diaphragm, abdominals, and accessory muscles (26). Images were processed offline on FIJI/ImageJ (National Institutes of Health, Bethesda, MD). Reliability of measuring diaphragm excursion via ultrasonography (ICC: intra-rate*r* = 0.93; inter-rate*r* = 0.88) was demonstrated in a previously published study (25).

### 2.8 Respiratory muscle quality

To obtain an index of respiratory muscle quality (MQ), we used an approach similar to what has been done for handgrip strength and forearm muscle thickness in the literature (27). For the respiratory muscles, MIP is a global assessment of voluntary motor output while thickness at FRC reflects diaphragm muscle mass at rest. As such, respiratory MQ was represented by the ratio of MIP (cmH_2_O) to diaphragm thickness at FRC (cm).

### 2.9 Exercise tolerance

Exercise tolerance was evaluated by the distance achieved in m on the 6-minute walk test (6MWT) according to ATS recommendations (28). Participants were instructed to walk as fast as possible along a 100-foot (30.48 m), out-and-back course for 6 min. Since 6MWT distance in children is highly dependent on age, sex, and anthropometrics, *z*-scores were calculated using reference equations specifically for children in order to normalize exercise tolerance to account for these differences (29).

### 2.10 Training protocol

IMT followed a protocol of inspiratory pressure threshold loading using the POWERbreathe Plus Inspiratory Muscle Trainer (POWERbreathe International Ltd., Warwickshire, UK). In pressure-threshold IMT, airflow is occluded until the generated inspiratory pressure reaches the predetermined threshold, which can be set at increments of 8 cmH_2_O. This type of IMT has been successfully used in several pediatric populations (30–33). During the first week of training, the target intensity was set at 50% of the baseline MIP. During the second week, the target intensity was set at 60% of the baseline MIP. Beginning at the third week and for the remainder of the training program, the target intensity was set at 75% of the participant's baseline MIP. For all intensities, the training volume was 5 sets of 6 breaths per day (total of 30 breaths per day). The frequency of training was 5 days per week, for a duration of 6 weeks.

Training began following baseline testing during the first visit. Participants and their accompanying caregivers were instructed on the performance of the required inspiratory maneuver via verbal explanation and visual demonstration of the following steps: 1) expiration to residual volume; 2) strong inspiratory effort with the IMT device at the specified resistance for 1–2 s; and 3) expiration without resistance. Training breaths were performed with a noseclip and in a sitting position with upright posture, back unsupported, and feet supported. Participants and caregivers were given written instructions for the IMT program as well as a training log to be completed for each day of training. At the end of the training program, adherence was recorded as the percentage of prescribed training breaths performed over the 6-week period.

### 2.11 Data analysis

Using Global Lung Initiative (GLI) 2022 equations (34), *z*-scores were calculated for FVC, FEV_1_, and FEV_1_/FVC. For MIP, percent of predicted values according to published equations specifically for children (35) were used for normalization. For spirometry, the best trial from each maneuver was selected for analysis. To better account for the variable and dynamic nature of the diaphragm and chest wall, for MIP, CWE, diaphragm ultrasonography, and MQ, all trials were included in the analysis.

### 2.12 Statistical analysis

Statistical analysis was performed using R Statistical Software version 4.3.1 (36). Significance was set at *P* ≤ 0.05 for all tests. Normality was assessed using the Shapiro–Wilks test and verified visually using histograms. Data were presented as medians and interquartile range (IQR) unless otherwise specified. Group comparisons for demographic and baseline data were made using Wilcoxon rank-sum tests.

To assess for the effects of IMT, linear mixed-effects models were constructed via the lmerTest package (37) using Group (CCS vs. HTD) and Time (T1 vs. T2) as fixed effects with participants as a random effect. MIP, CWE, diaphragm thickness/excursion, MQ, and exercise tolerance, were modeled as the response. Except for the 6MWT, trial number was also included as a random effect to account for the potential variability of those testing measures between trials and to improve the estimates of the fixed effects. Multiple *F*-tests were performed using Satterthwaite's method via the lmerTest package to evaluate the significance of model effects. Estimated marginal means, standardized effect sizes (Cohen's *d*), and the corresponding 95% confidence intervals (CI) were calculated using the emmeans package (38). Post-hoc, pairwise contrasts were made using Tukey's method of *P*-value adjustment.

## 3 Results

### 3.1 Participant characteristics

A total of 7 CCS [3 females and 4 males; median age=9 (3.5)] and 10 HTD [5 females and 5 males; median age=8 (2.5)] were enrolled into the study. In the CCS group (*n* = 7), five had a diagnosis of acute lymphoblastic leukemia, one had rhabdomyosarcoma, and one had Wilms tumor. The median time from the completion of cancer treatment was 4 years (IQR=1.5 years). At baseline, there were no between-group differences in participant characteristics except for greater exercise tolerance in HTD, according to both 6MWT distance in m [median in CCS=530.53 (34.21) vs. median in HTD=602.02 (100.84); *P* = 0.01] and 6MWT *z*-score [median in CCS=−1.61 (0.75) vs. median in HTD=0.07 (0.98); *P* = 0.03].

All but two participants completed the 6-week training program. One participant in the CCS group was lost to follow-up while one in the HTD group declined to continue training after week 5. Adherence to the training program was similar between groups. Of the participants who completed the 6-week training, all but one in the CCS group performed greater than 90% of the number of prescribed training breaths.

### 3.2 Effects of training

Overall, spirometry was not different after IMT in either group. There were no main effects or interactions of Time or Group on FVC, FEV_1_, and FEV_1_/FVC (*P* > 0.05 for all). However, there was a main effect of Time on MIP (% predicted) without interaction [main effect: *F*(1,74) = 35.61, *P* < 0.001]. *Post-hoc* contrasts showed that MIP (% predicted) was significantly higher after training in both groups [T1 vs. T2 in CCS: +35.14, 95% CI:(6.36, 63.93) %, *P* = 0.01, *d* = 1.27 (0.43, 2.12); T1 vs. T2 in HTD: +43.09 (23.06, 63.11) %, *P* < 0.001, *d* = 1.56 (0.93, 2.19)].

**Figure 1:**
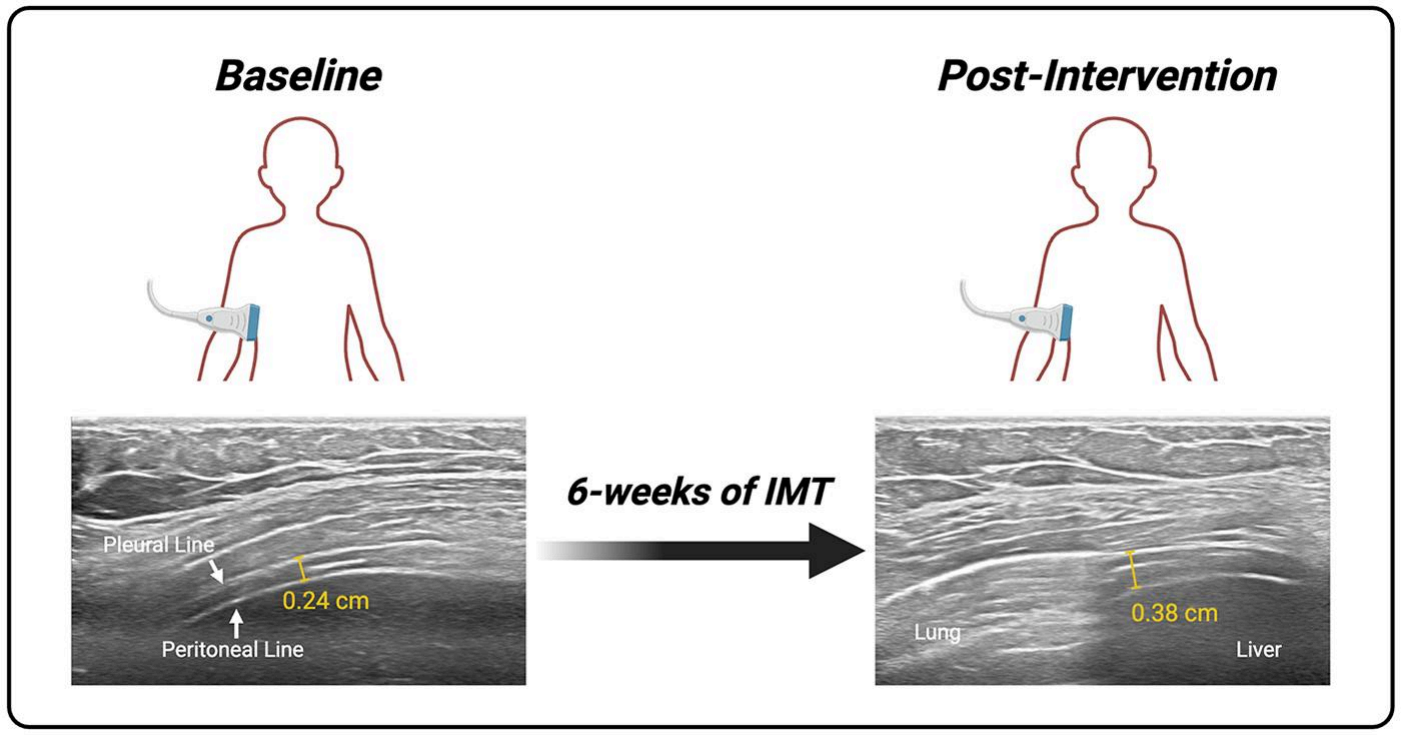
Example of B-mode ultrasonography obtained from the right costal diaphragm at the zone of apposition demonstrating the effects of IMT on diaphragm thickness. In B-mode, the pleural and peritoneal lines are clearly visible. Between these lines is a fibrous muscular layer representing the diaphragm. Thickness was defined as the perpendicular distance from the middle of the pleural line to the middle of the peritoneal line. Images show thickness at total lung capacity (TLC) in an 11-year-old, male childhood cancer survivor (CCS) at baseline (T1) and post-intervention (T2). Overall, thickness at TLC improved in the CCS group [T1 vs. T2 in CCS: +0.05 (0.01, 0.1) cm, *P* = 0.02], but not in healthy, typically developing, non-cancer controls. IMT, inspiratory muscle training. Created in BioRender. Ho, S. (2026) https://BioRender.com/la6v4q5.

The effects of IMT on diaphragm thickness were different for each group. There was no main effect of Time on thickness at TLC (*P* > 0.05), but there was an interaction of Time and Group [*F*(1,81) = 10.79, *P* = 0.002], suggesting that the two groups had opposite effects after training. *Post-hoc* contrasts showed that thickness at TLC increased significantly after training in the CCS group [T1 vs. T2 in CCS: +0.05 (0.01, 0.1) cm, *P* = 0.02, *d* = 1 (0.28, 1.71)] whereas there was a nonsignificant decrease in the HTD group [T1 vs. T2 in HTD: −0.02 (−0.06, 0.02) cm, *P* = 0.446, *d* = −0.41 (−0.98, 0.16)]. Furthermore, there was a main effect of Time on thickening fraction to TLC without interaction [main effect: *F*(1,83) = 7.52, *P* = 0.007]. *Post-hoc* contrasts showed a slight increase in both groups, with a trend for significance in the CCS group only [T1 vs. T2 in CCS: +0.28 (−0.02, 0.57), *P* = 0.071, *d* = 0.83 (0.13, 1.52)]. There were no differences in diaphragm excursion after training in either group (*P* > 0.05 for all). Figure 1 is an example of the increase in diaphragm thickness at TLC after IMT from a participant in the CCS group.

**Figure 2:**
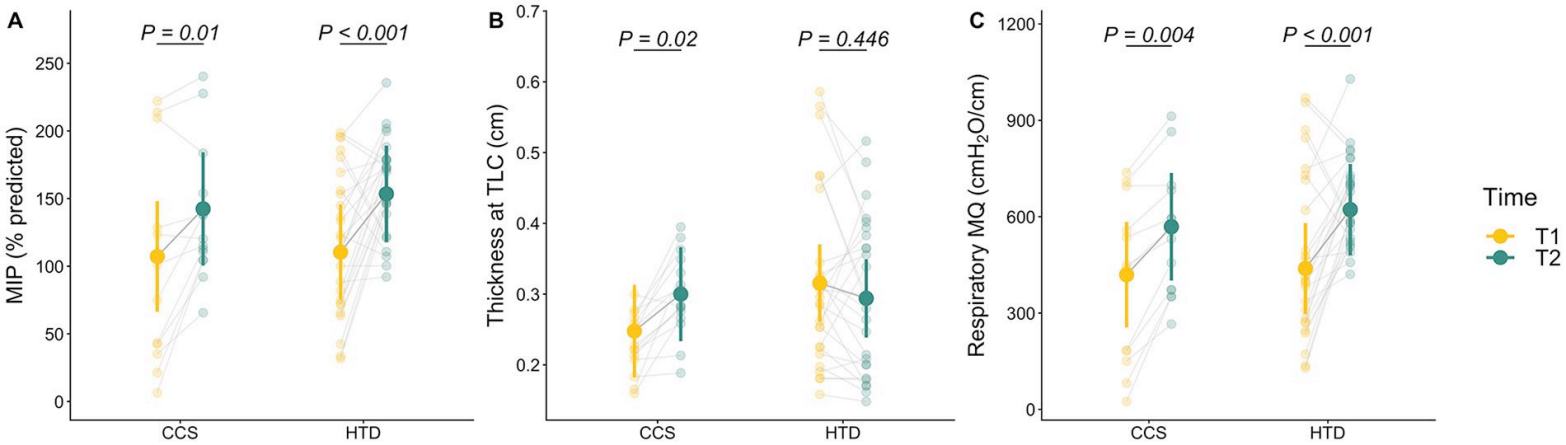
Effects of inspiratory muscle training on respiratory muscle performance. Individual observations are shown as semi-transparent points with a connecting line to show intra-individual change between time points. Solid points and accompanying vertical lines represent estimated marginal means and their 95% confidence intervals, respectively. **(A)**: MIP (% predicted) was significantly higher after training in both groups. There was a main effect of Time on MIP (% predicted) without interaction [main effect: F(1, 74) = 35.61, *P* < 0.001]. **(B)**: Thickness at TLC improved in CCS, but not in HTD. There was no main effect of Time on thickness at TLC (*P* > 0.05), but there was an interaction of Time and Group [*F*(1, 81) = 10.79, *P* = 0.002]. **(C)**: Respiratory MQ, which was determined by the ratio of MIP (cm H_2_O) to thickness at FRC (cm), improved in both groups. There was a main effect of Time on MQ without interaction [main effect: *F*(1, 74) = 42, *P* = < 0.001]. CCS, childhood cancer survivors; HTD: healthy, typically developing non-cancer controls; T1, before training; T2, after training; MIP, maximal inspiratory pressure; TLC, total lung capacity; MQ, muscle quality; FRC, functional residual capacity.

Respiratory MQ, which was determined by the ratio of MIP (cmH_2_O) to thickness at FRC (cm), improved after training in both groups. There was a main effect of Time on MQ without interaction [main effect: *F*(1,74) = 42, *P* = < 0.001]. *Post-hoc* contrasts showed that MQ was significantly higher after training in both groups [T1 vs. T2 in CCS: +149.64 (36.87, 262.41) cmH_2_O/cm, *P* = 0.004, *d* = 1.38 (0.53, 2.23); T1 vs. T2 in HTD: +183.22 (104.83, 261.61) cmH_2_O/cm, *P* < 0.001, *d* = 1.69 (1.06, 2.33)]. Figure 2 is a summary of the effects of IMT on respiratory muscle performance for each group.

**Figure 3:**
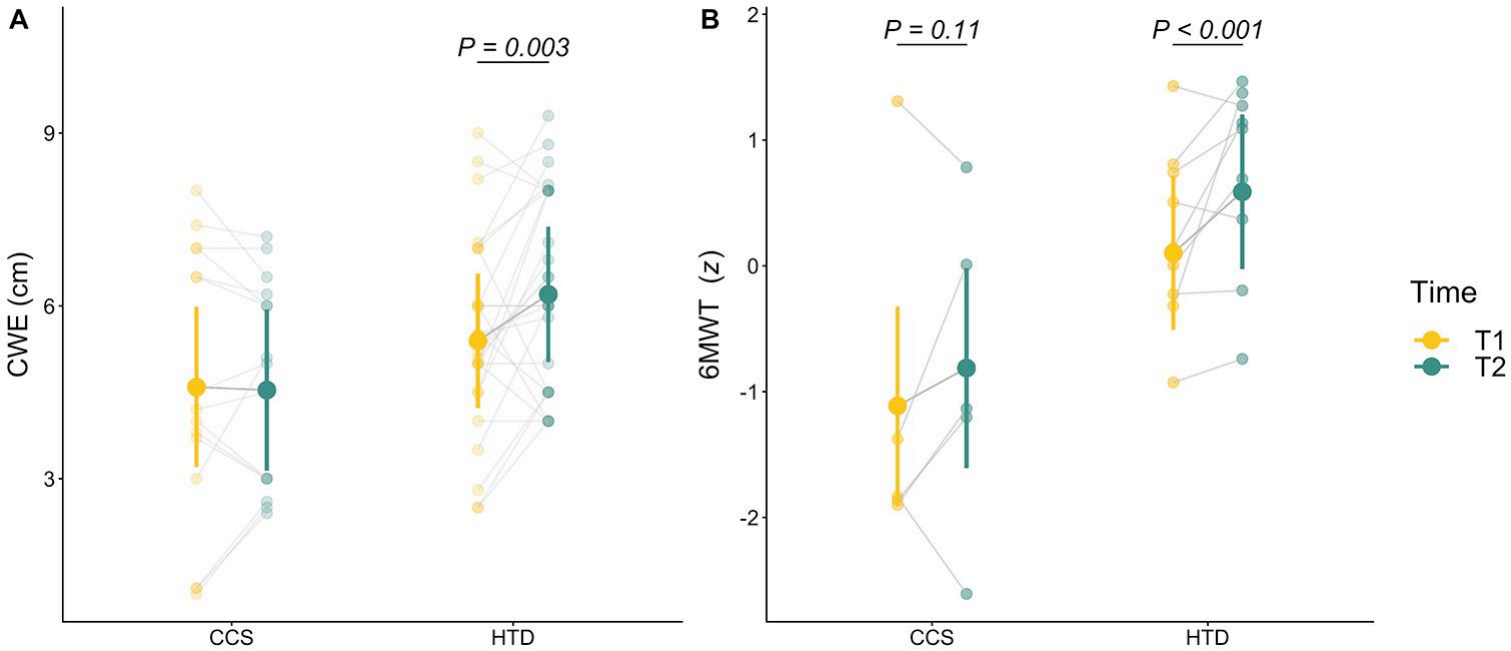
Effects of inspiratory muscle training on CWE and exercise tolerance. Individual observations are shown as semi-transparent points with a connecting line to show intra-individual change between time points. Solid points and accompanying vertical lines represent estimated marginal means and their 95% confidence intervals, respectively. **(A)**: CWE improved in HTD, but not in CCS. There was a main effect of Time and an interaction of Time and Group on CWE [main effect: *F*(1, 78) = 6.19, *P* = 0.015; interaction: *F*(1, 78) = 6.19, *P* = 0.015]. **(B)**: Exercise tolerance, measured by 6MWT *z*-score, also improved in HTD, but not in CCS. There was a main effect of Time on 6MWT *z*-score without interaction [main effect: *F*(1,75) = 23.67, *P* < 0.001]. CCS, childhood cancer survivors; HTD: healthy, typically developing non-cancer controls; T1, before training; T2, after training; CWE, chest wall excursion; 6MWT, 6-minute walk test.

The effects of IMT on CWE were dependent on group (Figure 3A). There was a main effect of Time and an interaction of Time and Group on CWE [main effect: *F*(1,78) = 6.19, *P* = 0.015; interaction: *F*(1,78) = 6.19, *P* = 0.015]. *Post-hoc* contrasts showed that CWE was significantly higher after training in the HTD group [T1 vs. T2 in HTD: +0.81 (0.23, 1.39) cm, *P* < 0.001, *d* = 1 (0.41, 1.6)] while there was a nonsignificant decrease in CWE in the CCS group [T1 vs. T2 in CCS: −0.05 (−0.76, 0.66) cm, *P* = 0.997, *d* = −0.07 (−0.77, 0.64)].

The effects of IMT on exercise tolerance, which was determined by the distance achieved on the 6MWT and its *z*-score, were also different for each group (Figure 3B). There was a main effect of Time on 6MWT distance in m without interaction [main effect: *F*(1,15) = 5.23, *P* = 0.038]. There was also a main effect of Time on 6MWT *z*-score without interaction [main effect: *F*(1,75) = 23.67, *P* < 0.001]. *Post-hoc* contrasts showed that the change in 6MWT distance was not significant after training in either group [T1 vs. T2 in HTD: +32.8 (−8.85, 74.45) m, *P* = 0.152, *d* = 1.14 (0.03, 2.24); T1 vs. T2 in CCS: +18.77 (−36.9, 74.44) m, *P* = 0.774, *d* = 0.65 (−0.77, 2.07)]. However, the *z*-score for 6MWT improved significantly after training in HTD [T1 vs. T2 in HTD: +0.49 (0.23, 0.74), *P* < 0.001, *d* = 0.85 (0.06, 1.63)], but not in CCS [T1 vs. T2 in CCS: +0.3 (−0.04, 0.65), *P* = 0.11, *d* = 1.37 (0.75, 1.98)].

## 4 Discussion

### 4.1 Main findings

The main finding of this exploratory study was that both CCS and HTD benefited from IMT, but in different ways. Although respiratory muscle performance, measured by MIP (% predicted) and MQ, improved in both groups, CWE improved in HTD, but not in CCS, whereas thickness at TLC improved in CCS, but not in HTD. Furthermore, exercise tolerance improved in HTD, but not in CCS. Thus, we provide preliminary findings of differential effects of IMT in each group, suggesting that there were differences in the response to training due to cancer treatment-related side effects in CCS. Despite these differences, the overall positive benefits of IMT support its clinical utility in CCS.

### 4.2 Implications of differential effects

The positive findings in respiratory muscle performance were consistent with previous studies that have used IMT in children (14, 33, 39) and in adult cancer survivors (22). What was unexpected was how each group responded to IMT to produce the observed benefits in respiratory muscle performance. We used a pressure-threshold device for IMT, which is known to create adaptations in pressure-generating capacity, but not necessarily flow and volume (40). During training, to overcome the external load placed by the device, inspiratory pressure would have to increase independent of flow. Over time, the expected adaptation would be either hypertrophy (41, 42) and/or increased muscle contractility (43). Therefore, an increase in diaphragm thickness as a response to training was expected in both groups. Whereas improvements in CWE and exercise tolerance were expected as secondary effects related to changes in diaphragm and chest wall mechanics and their interactions.

We were unable to detect improvements in diaphragm thickness in HTD possibly because the threshold for hypertrophy was not met by the prescribed IMT. Using a paradigm of high resistance and low volume (5 sets of 6 breaths at 75% of MIP), the training parameters should have been adequate to induce hypertrophy (42, 44, 45). However, because of the home-based nature of the training and the virtual follow-up, we did not perform measurements of MIP each week. Thus, all training parameters were based on baseline MIP. As such, by the third week, when participants progressed to performing training at 75% of MIP, their MIP likely improved to a new baseline, which meant that the training intensity might have been too low. Conversely, we were able to detect improved thickness in CCS likely because of lower muscle mass and function at baseline. As such, the required stimulus for hypertrophy was likely lower compared to what was required for HTD. In fact, the improvement in thickness observed in CCS might have represented “catch-up” growth. Although not statistically significant, baseline thickness at TLC in CCS tended to be lower compared to HTD. After IMT, thickness at TLC increased to levels similar to those found in HTD. In spite of these individual trends, diaphragm contractility likely improved in both groups, which would explain the improvement in MIP and MQ. However, since we did not measure transdiaphragmatic pressure, which is the gold-standard (46), this should be interpreted cautiously. Nonetheless, our findings suggest that the unique adaptations that took place in each group independently led to improvements in respiratory muscle performance.

The improvements in CWE found in HTD were likely the result of improved chest wall mechanics owing to more efficient respiratory muscles. On the other hand, the lack of improvements in CCS might have been attributed to the persistence of cancer treatment-related pulmonary dysfunction. Indeed, CCS are at an increased risk for pulmonary dysfunction and chest wall restrictions (5). Even with normal spirometry, pulmonary dysfunction could still be present and often remains undetected (3). As such, in the setting of a less compliant respiratory system and/or impaired gas exchange, the expected benefits from pressure-threshold IMT likely had a lesser impact on CCS. This could have partially explained why exercise tolerance did not improve in CCS. Hence, even though there were improvements in respiratory muscle performance and diaphragm thickness, CCS had limited benefits in gas exchange from IMT. Although underlying pulmonary dysfunction is a plausible explanation, this remains unconfirmed. This represents an important area for future investigations and will require more sensitive measurements such as diffusion capacity or ventilation heterogeneity to further evaluate possible mechanisms for why improvements in respiratory muscle performance did not translate into functional gains in CCS.

### 4.3 Clinical considerations

Although our findings suggest that that CCS were generally less responsive to IMT compared to HTD, we nevertheless demonstrated that respiratory MQ improved after IMT, which has not been done before in pediatric populations. By measuring the change in the diaphragm's strength relative to its size, we accounted for the effects of IMT on both muscle structure and function—thus further supporting that there were clinical benefits to this type of training. These muscle-specific findings were also clinically important because we were able to show that CCS, who are at high risk of cancer treatment-related muscle dysfunction (6), were responsive to exercise training that targeted skeletal muscle. However, the improvements in respiratory MQ should not be over-interpreted since MIP, which was used to derive this metric, reflects global inspiratory effort and does not solely point to changes in the diaphragm. Nonetheless, IMT appears to be a promising, nonpharmacological intervention strategy that can help mitigate the various late effects of cancer treatment.

### 4.4 Limitations

A potential limitation of this exploratory study was that the training intensity might have been too low, especially for the HTD group, since dosing was not adjusted based on weekly testing of MIP. This likely decreased the relative workload over the 6 weeks of training. Although the optimal dose and schedule for IMT in children remains unclear, future studies should reassess MIP weekly in order to maintain the desired training intensity. In addition, training adherence was recorded via self-report, which is a limitation. Future studies should consider using more accurate methods to track progress, such as the use of electronic IMT devices or smartphone applications that can automatically log training sessions. As such, much work is still needed to optimize this intervention for widespread clinical adoption.

We also assumed that diaphragm contractility improved after IMT because of improvements in MIP and MQ, which should be cautiously interpreted. However, the gold standard for this measurement is still transdiaphragmatic pressure, which is highly invasive (46). Nonetheless, several non-invasive, advanced imaging methods, such as strain analysis via speckle-tracking (47) and ultrafast ultrasound (48), show great promise in objectively quantifying contractility and can be used in future studies.

Furthermore, ultrasound imaging in our study was not blinded, which could have introduced bias that would impact the interpretation of changes in diaphragm thickness and thus hypertrophy. As such, the positive findings for this outcome in CCS should be considered preliminary and should be interpreted with caution. Future studies should ensure that assessors are blinded, using separate personnel to deliver training and for outcomes assessment. Other strategies include using separate assessors for image acquisition and processing as well as blinding the assessors during offline image processing to when images were obtained (i.e., baseline vs. post-intervention).

Finally, there were some sampling considerations. The small sample size in this exploratory study limits statistical power, especially for functional outcomes such as the 6MWT. In addition, we used a convenience sample of CCS and we did not have detailed cancer treatment data, thus making clinical interpretation more challenging. For instance, CCS exposed to cancer treatments that are known to increase the risk of muscle dysfunction, such as anthracyclines (7), may respond differently to resistance training. Similarly, those exposed to therapies with known pulmonary toxicity, such as bleomycin and busulfan (4), could potentially experience differential effects to IMT because of impaired lung capacity. However, without specific data on prior therapy, we were unable to assess the impact of cancer treatment exposure or the severity of late effects. Future studies with a larger sample of CCS with detailed cancer treatment exposures will be needed in order to delineate the effects of IMT on CCS with different risk factors and late effects.

### 4.5 Future directions

Since the lack of improvement in CWE was likely a limiting factor that was specific to CCS, flow-resistive IMT could be a useful alternative or adjunct to pressure-threshold IMT. This is because flow-resistive IMT would improve flow rate (40), potentially allowing for more rapid filling of the chest and thus a better ability to quickly increase ventilation, especially in response to exercise. Thus, the addition of flow-resistive IMT could potentially fill a gap in the overall benefits of IMT left unaddressed by pressure-threshold IMT, specifically for CCS. However, the effects of flow-resistive IMT in CCS are not known. Future studies should compare the effects of using both pressure-threshold and flow-resistive IMT vs. either one alone. Furthermore, a deeper understanding of how each group adapted to IMT would be needed in order to optimize training in future studies. The addition of more advanced imaging modalities, such as shear wave elastography (49), could provide important insights on the effects of IMT on the mechanical properties of the diaphragm.

## 5 Conclusions

In summary, the strongest finding from this pilot study was that pressure-threshold IMT improved respiratory muscle performance in both CCS and HTD. In addition, we present preliminary findings of differential effects from IMT on chest wall mechanics and exercise tolerance between the two groups. Surprisingly, diaphragm thickness improved in CCS, but not in HTD. Whereas CWE and exercise tolerance improved in HTD, but not in CCS. The lack of improvement in CWE was possibly a limiting factor for CCS, as it coincided with a lack of improvement in exercise intolerance. The trends for CCS might perhaps point to persistent pulmonary dysfunction or other late effects such as cardiac or vascular dysfunction. However, this explanation will have to be confirmed in the future given the exploratory nature of our study. Nonetheless, both groups ultimately benefited from the intervention—supporting the clinical utility of IMT. Future work using larger sample sizes and randomized designs will be needed to establish clinical efficacy and to optimize training parameters for CCS.

## Data Availability

The raw data supporting the conclusions of this article will be made available by the authors, without undue reservation.

## References

[1] Sultan I Alfaar AS Sultan Y Salman Z Qaddoumi I. Trends in childhood cancer: incidence and survival analysis over 45 years of SEER data. PLoS One. (2025) 20(1):e0314592. 10.1371/journal.pone.0314592 39752445 PMC11698462

[2] Suh E Stratton KL Leisenring WM Nathan PC Ford JS Freyer DR Late mortality and chronic health conditions in long-term survivors of early-adolescent and young adult cancers: a retrospective cohort analysis from the Childhood Cancer Survivor Study. Lancet Oncol. (2020) 21(3):421–35. 10.1016/S1470-2045(19)30800-9 32066543 PMC7392388

[3] Schindera C Usemann J Zuercher SJ Jung R Kasteler R Frauchiger B Pulmonary dysfunction after treatment for childhood cancer. Comparing multiple-breath washout with spirometry. Ann Am Thorac Soc. (2021) 18(2):281–9. 10.1513/AnnalsATS.202003-211OC 32877212

[4] Otth M Kasteler R Mulder RL Agrusa J Armenian SH Barnea D Recommendations for surveillance of pulmonary dysfunction among childhood, adolescent, and young adult cancer survivors: a report from the International Late Effects of Childhood Cancer Guideline Harmonization Group. EClinicalMedicine. (2024) 69:102487. 10.1016/j.eclinm.2024.102487 38420219 PMC10900250

[5] Huang TT Hudson MM Stokes DC Krasin MJ Spunt SL Ness KK. Pulmonary outcomes in survivors of childhood cancer: a systematic review. Chest. (2011) 140(4):881–901. 10.1378/chest.10-2133 21415131 PMC3904488

[6] Goodenough CG Partin RE Ness KK. Skeletal muscle and childhood cancer: where are we now and where we go from here. Aging Cancer. (2021) 2(1-2):13–35. 10.1002/aac2.12027 34541550 PMC8445321

[7] Tarpey MD Amorese AJ Balestrieri NP Fisher-Wellman KH Spangenburg EE. Doxorubicin causes lesions in the electron transport system of skeletal muscle mitochondria that are associated with a loss of contractile function. J Biol Chem. (2019) 294(51):19709–22. 10.1074/jbc.RA119.008426 31690631 PMC6926446

[8] Campelj DG Goodman CA Rybalka E. Chemotherapy-induced myopathy: the dark side of the cachexia sphere. Cancers (Basel). (2021) 13(14):3615. 10.3390/cancers13143615 34298829 PMC8304349

[9] Gilliam LAA Moylan JS Callahan LA Sumandea MP Reid MB. Doxorubicin causes diaphragm weakness in murine models of cancer chemotherapy. Muscle Nerve. (2011) 43(1):94–102. 10.1002/mus.21809 21171100 PMC3057655

[10] Ness KK Plana JC Joshi VM Luepker RV Durand JB Green DM Exercise intolerance, mortality, and organ system impairment in adult survivors of childhood cancer. J Clin Oncol. (2020) 38(1):29–42. 10.1200/JCO.19.01661 31622133 PMC7051850

[11] Ho S Betz G Marchese V. Exploring pulmonary function and physical function in childhood cancer: a systematic review. Crit Rev Oncol Hematol. (2021) 160:103279. 10.1016/j.critrevonc.2021.103279 33716200

[12] Lanfranconi F Zardo W Moriggi T Villa E Radaelli G Radaelli S Precision-based exercise as a new therapeutic option for children and adolescents with haematological malignancies. Sci Rep. (2020) 10(1):12892. 10.1038/s41598-020-69393-1 32733066 PMC7393502

[13] Fridh MK Schmidt-Andersen P Andrés-Jensen L Thorsteinsson T Wehner PS Hasle H Children with cancer and their cardiorespiratory fitness and physical function-the long-term effects of a physical activity program during treatment: a multicenter non-randomized controlled trial. J Cancer Surviv. (2025) 19(2):672–84. 10.1007/s11764-023-01499-7 38057671 PMC11926049

[14] Bhammar DM Jones HN Lang JE. Inspiratory muscle rehabilitation training in pediatrics: what is the evidence? Can Respir J. (2022) 2022:5680311. 10.1155/2022/5680311 36033343 PMC9410970

[15] Gawade PL Hudson MM Kaste SC Neglia JP Wasilewski-Masker K Constine LS A systematic review of selected musculoskeletal late effects in survivors of childhood cancer. Curr Pediatr Rev. (2014) 10(4):249–62. 10.2174/1573400510666141114223827 25403639 PMC4336580

[16] Khan F Williams AM Weiner DJ Constine LS. Impact of respiratory developmental stage on sensitivity to late effects of radiation in pediatric cancer survivors. Adv Radiat Oncol. (2020) 5(3):426–33. 10.1016/j.adro.2019.12.002 32529137 PMC7276690

[17] Urakawa R Noi A Kageyama H Ueda M Hashii Y Ikeda K. Association between treatment of childhood cancer and its late effects on gonadal or growth function in childhood cancer survivors: a retrospective observational study. Cancer Med. (2025) 14(6):e70805. 10.1002/cam4.70805 40123531 PMC11931328

[18] Nguyen BL Baumfalk DR Lapierre-Nguyen SS Zhong R Doerr V Montalvo RN Effects of exercise and doxorubicin on acute diaphragm neuromuscular transmission failure. Exp Neurol. (2024) 378:114818. 10.1016/j.expneurol.2024.114818 38782352 PMC11616575

[19] Mendonca CT Schaeffer MR Riley P Jensen D. Physiological mechanisms of dyspnea during exercise with external thoracic restriction: role of increased neural respiratory drive. J Appl Physiol (1985). (2014) 116(5):570–81. 10.1152/japplphysiol.00950.2013 24356524 PMC3949213

[20] D'Lugos AC Fry CS Ormsby JC Sweeney KR Brightwell CR Hale TM Chronic doxorubicin administration impacts satellite cell and capillary abundance in a muscle-specific manner. Physiol Rep. (2019) 7(7):e14052. 10.14814/phy2.14052 30963722 PMC6453819

[21] Moreira-Pais A Ferreira R Baltazar T Neuparth MJ Vitorino R Reis-Mendes A Long-term effects of the chronic administration of doxorubicin on aged skeletal muscle: an exploratory study in mice. Biochem Biophys Res Commun. (2024) 733:150650. 10.1016/j.bbrc.2024.150650 39255618

[22] Ray AD Williams BT Mahoney MC. Respiratory muscle training improves exercise performance and quality of life in cancer survivors: a pilot study. Rehabil Oncol. (2017) 35(2):81–9. 10.1097/01.REO.0000000000000064

[23] Graham BL Steenbruggen I Miller MR Barjaktarevic IZ Cooper BG Hall GL Standardization of spirometry 2019 update. An official American Thoracic Society and European Respiratory Society technical statement. Am J Respir Crit Care Med. (2019) 200(8):e70–88. 10.1164/rccm.201908-1590ST 31613151 PMC6794117

[24] Laveneziana P Albuquerque A Aliverti A Babb T Barreiro E Dres M ERS Statement on respiratory muscle testing at rest and during exercise. Eur Respir J. (2019) 53(6):1801214. 10.1183/13993003.01214-2018 30956204

[25] Ho S Rock K Addison O Marchese V. Relationships between diaphragm ultrasound, spirometry, and respiratory mouth pressures in children. Respir Physiol Neurobiol. (2022) 305:103950. 10.1016/j.resp.2022.103950 35905862

[26] Katagiri M Abe T Yokoba M Dobashi Y Tomita T Easton PA. Neck and abdominal muscle activity during a sniff. Respir Med. (2003) 97(9):1027–35. 10.1016/s0954-6111(03)00133-1 14509557

[27] Abe T Thiebaud RS Loenneke JP. Age-related change in handgrip strength in men and women: is muscle quality a contributing factor? Age (Dordr). (2016) 38(1):28. 10.1007/s11357-016-9891-4 26874950 PMC5005880

[28] ATS Committee on Proficiency Standards for Clinical Pulmonary Function Laboratories. ATS statement: guidelines for the six-minute walk test. Am J Respir Crit Care Med. (2002) 166(1):111–7. 10.1164/ajrccm.166.1.at1102 12091180

[29] Saraff V Schneider J Colleselli V Ruepp M Rauchenzauner M Neururer S Sex-, age-, and height-specific reference curves for the 6-min walk test in healthy children and adolescents. Eur J Pediatr. (2015) 174(6):837–40. 10.1007/s00431-014-2454-8 25491900

[30] Wu FM Opotowsky AR Denhoff ER Gongwer R Gurvitz MZ Landzberg MJ A pilot study of inspiratory muscle training to improve exercise capacity in patients with Fontan physiology. Semin Thorac Cardiovasc Surg. (2018) 30(4):462–9. 10.1053/j.semtcvs.2018.07.014 30063966

[31] Zeren M Cakir E Gurses HN. Effects of inspiratory muscle training on postural stability, pulmonary function and functional capacity in children with cystic fibrosis: a randomised controlled trial. Respir Med. (2019) 148:24–30. 10.1016/j.rmed.2019.01.013 30827470

[32] Silva IS Pedrosa R Azevedo IG Forbes AM Fregonezi GA Junior D Respiratory muscle training in children and adults with neuromuscular disease. Cochrane Database Syst Rev. (2019) 9(9):CD011711. 10.1002/14651858.CD011711.pub2 31487757 PMC6953358

[33] Lin CH Lee CW Huang CH. Inspiratory muscle training improves aerobic fitness in active children. Int J Environ Res Public Health. (2022) 19(22):14722. 10.3390/ijerph192214722 36429439 PMC9690705

[34] Bowerman C Bhakta NR Brazzale D Cooper BR Cooper J Gochicoa-Rangel L A race-neutral approach to the interpretation of lung function measurements. Am J Respir Crit Care Med. (2023) 207(6):768–74. 10.1164/rccm.202205-0963OC 36383197

[35] Verma R Chiang J Qian H Amin R. Maximal static respiratory and sniff pressures in healthy children. A systematic review and meta-analysis. Ann Am Thorac Soc. (2019) 16(4):478–87. 10.1513/AnnalsATS.201808-506OC 30562038

[36] R Core Team. R: A Language and Environment for Statistical Computing. Vienna, Austria: R Foundation for Statistical Computing (2022). Available online at: https://www.R-project.org/ (Accessed January 23, 2023).

[37] Kuznetsova A Brockhoff PB Christensen RHB. lmerTest package: tests in linear mixed effects models. J Stat Soft. (2017) 82(13):1–26. 10.18637/jss.v082.i13

[38] Lenth RV. Emmeans: Estimated Marginal Means, aka Least-Squares Means. (2017) 10.32614/CRAN.package.emmeans (Accessed March 31, 2025).

[39] Kaeotawee P Udomittipong K Nimmannit A Tovichien P Palamit A Charoensitisup P Effect of threshold inspiratory muscle training on functional fitness and respiratory muscle strength compared to incentive spirometry in children and adolescents with obesity: a randomized controlled trial. Front Pediatr. (2022) 10:942076. 10.3389/fped.2022.942076 35874588 PMC9302609

[40] Romer LM McConnell AK. Specificity and reversibility of inspiratory muscle training. Med Sci Sports Exerc. (2003) 35(2):237–44. 10.1249/01.MSS.0000048642.58419.1E 12569211

[41] Enright SJ Unnithan VB Heward C Withnall L Davies DH. Effect of high-intensity inspiratory muscle training on lung volumes, diaphragm thickness, and exercise capacity in subjects who are healthy. Phys Ther. (2006) 86(3):345–54. 10.1093/ptj/86.3.345 16506871

[42] Mills DE Johnson MA Barnett YA Smith WHT Sharpe GR. The effects of inspiratory muscle training in older adults. Med Sci Sports Exerc. (2015) 47(4):691–7. 10.1249/MSS.0000000000000474 25116085

[43] Ramsook AH Molgat-Seon Y Schaeffer MR Wilkie SS Camp PG Reid WD Effects of inspiratory muscle training on respiratory muscle electromyography and dyspnea during exercise in healthy men. J Appl Physiol (1985). (2017) 122(5):1267–75. 10.1152/japplphysiol.00046.2017 28255085 PMC5451532

[44] Haun CT Vann CG Osburn SC Mumford PW Roberson PA Romero MA Muscle fiber hypertrophy in response to 6 weeks of high-volume resistance training in trained young men is largely attributed to sarcoplasmic hypertrophy. PLoS One. (2019) 14(6):e0215267. 10.1371/journal.pone.0215267 31166954 PMC6550381

[45] Vann CG Sexton CL Osburn SC Smith MA Haun CT Rumbley MN Effects of high-volume versus high-load resistance training on skeletal muscle growth and molecular adaptations. Front Physiol. (2022) 13:857555. 10.3389/fphys.2022.857555 35360253 PMC8962955

[46] American Thoracic Society/European Respiratory Society. ATS/ERS Statement on respiratory muscle testing. Am J Respir Crit Care Med. (2002) 166(4):518–624. 10.1164/rccm.166.4.518 12186831

[47] Glau CL Lin EE Conlon TW Himebauch AS Keim GP Nishisaki A. Ultrasound assessment of diaphragm thickness, contractility, and strain in healthy pediatric patients. Pediatr Pulmonol. (2024) 59(2):433–41. 10.1002/ppul.26768 38038168 PMC11810524

[48] Poulard T Dres M Niérat MC Rivals I Hogrel JY Similowski T Ultrafast ultrasound coupled with cervical magnetic stimulation for non-invasive and non-volitional assessment of diaphragm contractility. J Physiol. (2020) 598(24):5627–38. 10.1113/JP280457 32997791

[49] Sinanoğlu MS Güngör Ş Dağ N Varol Fİ Kenç Ş Gök E. Ultrasound and shear wave elastography assessment of diaphragm thickness and stiffness in malnourished pediatric patients. Eur J Pediatr. (2024) 184(1):35. 10.1007/s00431-024-05852-5 39567388

